# Supplementary material for: Enhanced Enantioselective Optical Trapping Enabled by Longitudinal Mie Resonances in Silicon Nanodisks
Source: Nanophotonics. 2026 Jul 18;15(14):e70207. doi: 10.1002/nap2.70207 (PMC13642093; doi:10.1002/nap2.70207)
Supplement: Supplementary file 1 [file NAP2-15-e70207-s001.pdf]

Supporting Information for

Enhanced Enantioselective Optical Trapping enabled by Longitudinal Mie Resonances in Silicon Nanodisks

Guillermo Serrera Pablo Albella**

Group of Optics, Department of Applied Physics, University of Cantabria, 39005, Spain
Email Address: guillermo.serrera@unican.es; pablo.albella@unican.es

Keywords: *Optical manipulation, optical forces, nano-optical tweezers, enantiomer separation, chiral nanoparticles*

S1. Full paraxial ARPB expression

Equation 1 in the main text reads

$$\mathbf{E}^{\mathbf{ARPB}} = \mathbf{E}^{\mathbf{APB}} + \mathbf{E}^{\mathbf{RPB}} = \frac{V_R}{w^2} f(A_\rho + iB_\rho)\hat{\boldsymbol{\rho}} + \frac{V_A}{w^2} f e^{i\Psi}\hat{\boldsymbol{\varphi}} + \frac{2iV_R}{kw^2} f(A_z + iB_z)\hat{\mathbf{z}} \tag{S1}$$

where V_R and V_A are volt-unit amplitudes for the radial and azimuthal polarizations and Ψ is a constant phase difference between them [1]. $w = w_0\sqrt{1+(z/z_R)^2}$ is the beam radius, which at the focus point is equal to w_0; and $z_R = \pi w_0^2/\lambda$ is the Rayleigh range of the beam. The shorthand A and B parameters, plus the Gaussian envelope f, are given by

$$A_\rho = 1 + \frac{1}{kz_R}\frac{\rho^2 - 2w_0^2}{w^2} + \left(\frac{2z\rho}{w^2 kz_R}\right)^2 \tag{S2a}$$

$$B_\rho = -\frac{4}{k^2w^2}\frac{z}{z_R}\left(1 - \frac{\rho^2}{w^2}\right) \tag{S2b}$$

$$A_z = 1 - \frac{\rho^2}{w^2} \tag{S2c}$$

$$B_z = \frac{z}{z_R}\frac{\rho^2}{w^2} \tag{S2d}$$

$$f = \frac{2}{\sqrt{\pi}} e^{-\left(\frac{\rho}{w}\right)^2\zeta - 2i\tan^{-1}\left(\frac{z}{z_R}\right) + ikz} \tag{S2e}$$

with k being the wavenumber and $\zeta = 1 - iz/z_R$ being the Gouy phase. The total power of the beam, in a medium with impedance η, is given by

$$P_{ARPB} = P_{APB} + P_{RPB} = \frac{1}{2\eta}\left(|V_A|^2 + |V_R|^2\right)\left(1 - \frac{1}{\omega z_R}\right) \tag{S3}$$

S2. Cartesian multipole components

The scattered powers depicted in Figures 1a, 1b and 1c in the main document come from the computation of the multipolar moment tensors from the internal electric fields, as shown in [2]. Figure S1 contains a decomposition of the cartesian components of the different multipolar moment tensors, allowing us to discern whether the excited resonances are truly of longitudinal nature.

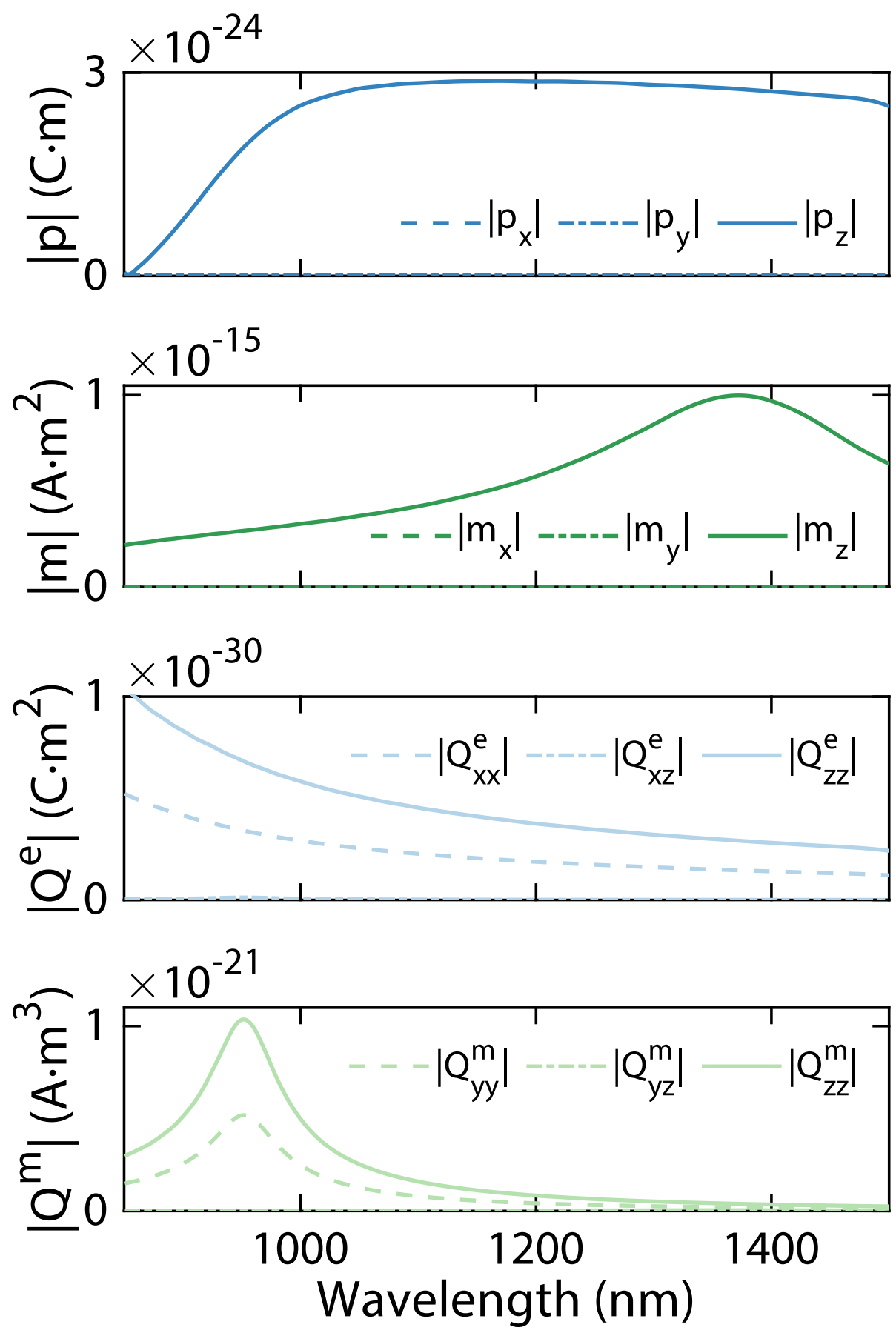

Figure S1: Absolute value of the different Cartesian components of the multipole moment tensors in Figure 1c of the main document. For dipolar resonances, characterized by first-rank tensors (vectors), all three components are depicted. For quadrupolar resonances, characterized by second-rank tensors, only the three independent components (diagonal xx/yy, diagonal zz, and off-diagonal xz/yz) are featured.

For first-order resonances (electric and magnetic dipoles, corresponding to first-rank tensors), Figure S1 contains all three elements (x, y and z), with the longitudinal z component being the only relevant contributions.

On the other hand, quadrupolar resonances (corresponding to second-rank tensors) display two relevant contributions. While the longitudinal zz elements are still the largest contributors, other diagonal tensor elements are also important. Off-diagonal terms, often the dominant contributors with plane wave illuminations, are negligible with ARPB illumination. It should be noted that, due to the cylindrical symmetry of the system, $|Q^{e,m}_{xx}| = |Q^{e,m}_{yy}|$ and $|Q^{e,m}_{xz}| = |Q^{e,m}_{zx}| = |Q^{e,m}_{zy}| = |Q^{e,m}_{yz}|$, so Figure S1 shows all independent components of the tensors.

1 S3. Dissipative and spin density force contributions

As noted in the main document, the force in its equation 3 can be separated into conservative reactive forces, depending on the electric field intensity and optical chirality gradients:

$$\langle \mathbf{F} \rangle_{reac} = \frac{1}{4}\mathfrak{Re}(\alpha_e)\nabla|\mathbf{E}|^2 - \frac{1}{2}\mathfrak{Re}(\alpha_{em})\nabla\left[\mathfrak{Im}(\mathbf{E}^* \cdot \mathbf{H})\right] \tag{S4}$$

and dissipative forces, coming from scattering and the electric field spin-density.

$$\langle \mathbf{F} \rangle_{diss} = \sigma \frac{\langle \mathbf{S} \rangle}{c} + \omega \gamma_e \langle \mathbf{L_e} \rangle \tag{S5}$$

Integration of forces to compute a potential is only possible if these dissipative forces are negligible against the reactive ones. To check that this condition is met, a decomposition of these force terms is shown in Figure S2 for the two wavelengths analyzed in Figures 1 and 2 of the main document. For the magnetic dipole resonance wavelength (1350 nm), it is clear that the x component of the reactive force is restorative, pushing objects to the center of the disk's upper face, while the stronger z component pushes the object towards the upper face. This is also observed at the magnetic quadrupole resonance wavelength (950 nm), where the reactive x component has two equilibrium points, corresponding to the potential wells observed in Figure 2. For both wavelengths, the reactive y component (evaluated at the xz plane) is exactly zero due to the cylindrical symmetry of the system.

More importantly, the scattering and spin dissipative terms of the force are in all cases negligible in comparison with the reactive terms, enabling us to compute potentials from the reactive terms. In particular, while reactive forces are often in the 0.01-0.1 pN range, scattering and spin forces are around five orders of magnitude smaller.

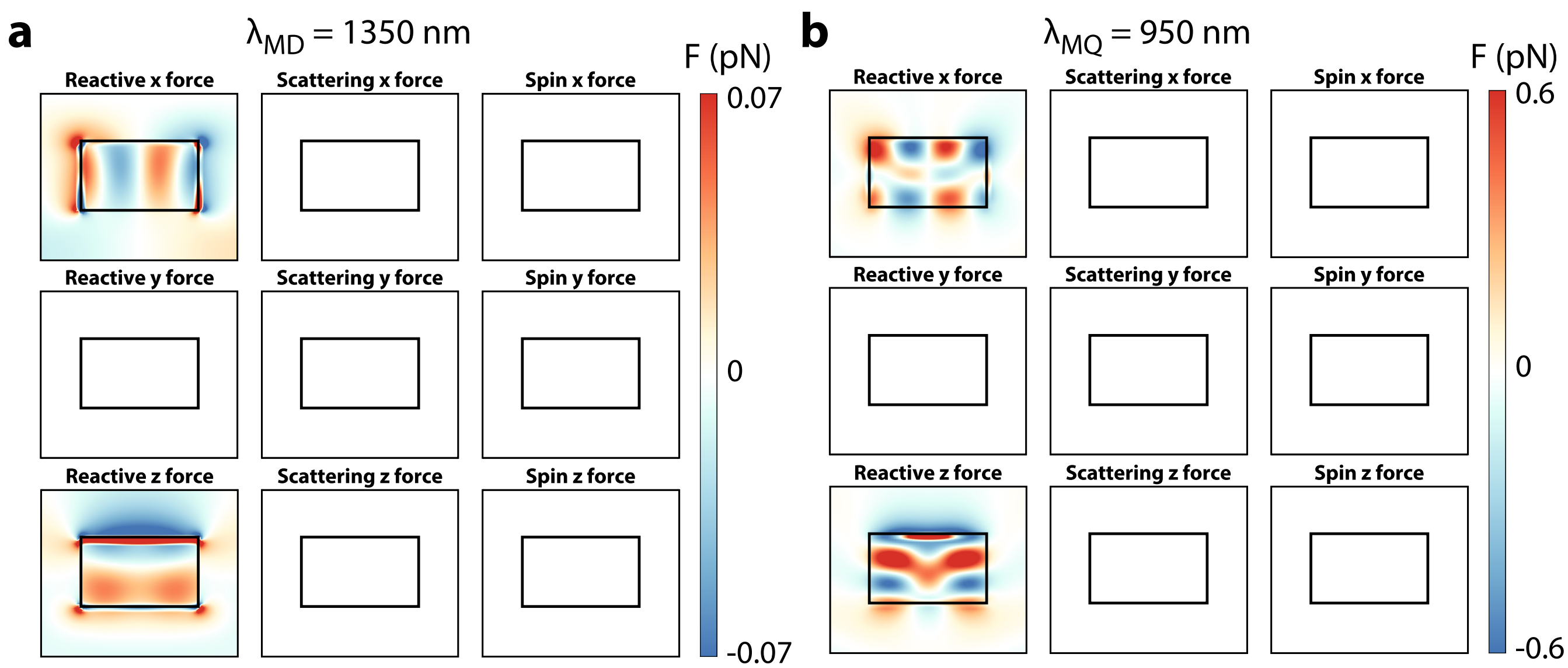

Figure S2: Decomposition of the forces displayed in Figure 2 of the main document in their cartesian components and reactive and dissipative (scattering and spin) terms.

S4. Details of potential fitting

As stated in the methods section of the main document, potentials were calculated by numerical integration of the forces $U_{\rho z} = \int F_\rho d\rho + \int F_z dz$. Integration in the radial domain was performed by considering the $\rho = 400$ nm endpoint shown in Figure 4d as a zero potential point, while integration in the z direction was considered only from the upper face level to 200 nm above, which was fixed as a zero potential level as well. To compute escape times from the potential curvatures, we fit the bistable radial potential profiles to a quartic analytical form $U(\rho) = a\rho^4 + b\rho^2 + c$ in the $[0, 150]$ nm radial interval [3]. This interval allows to capture most of the disk's upper face, while leaving outside the sharp potential spikes, which correspond to lightning rod effects. As shown for exemplary cases in Figure S3, the fit within this interval is very good, and allows to reproduce potential minima even at the weakest focusing ($NA = 0.3$), where the bistable minima are well resolved.

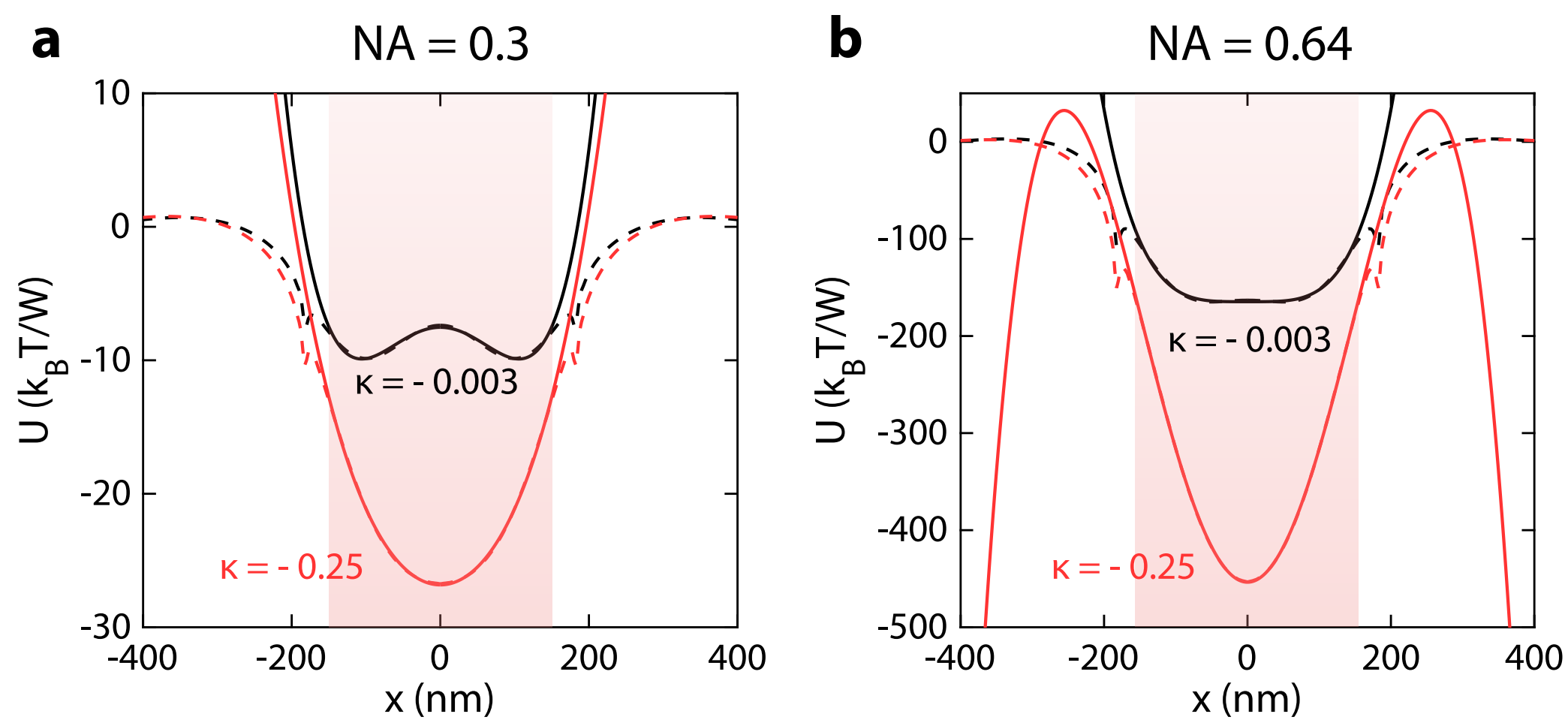

Figure S3: In dashed lines, radial profile of some of the potentials at the disk upper face, with the quartic fitting in solid lines. The fitting is only performed in the $[-150, 150]$ nm interval, represented by the reddish region in the background. a) Potential with $NA = 0.3$ illumination ($\kappa = -0.003$ in black, $\kappa = -0.25$ in red). b) Potential with $NA = 0.64$ illumination ($\kappa = -0.003$ in black, $\kappa = -0.25$ in red).

Such a fit allows to easily detect the potential minima and extract their curvature $U''(x_0)$ could be easily extracted. For strong positive values of κ, the potential might not have a minimum in this range. This situation corresponds to a repulsion, so if no minima could be found, the curvature was not computed and instead the mean escape time was set to zero and thus an infinite selectivity.

On the other hand, the escape point curvatures $|U''(x_{esc})|$ were obtained from numerical derivatives:

$$U''(x_{esc}) \approx \frac{U(x_{esc} + \Delta x) + U(x_{esc} - \Delta x) - 2U(x_{esc})}{\Delta x^2} \tag{S6}$$

The escape points were determined by finding the potential maxima beyond the disk radius ($\rho \geq 200$ nm). In this case, a repulsion situation results in no maxima found in this range. Likewise, the curvature was not computed in those cases, resulting again in a zero mean escape time and an infinite selectivity.

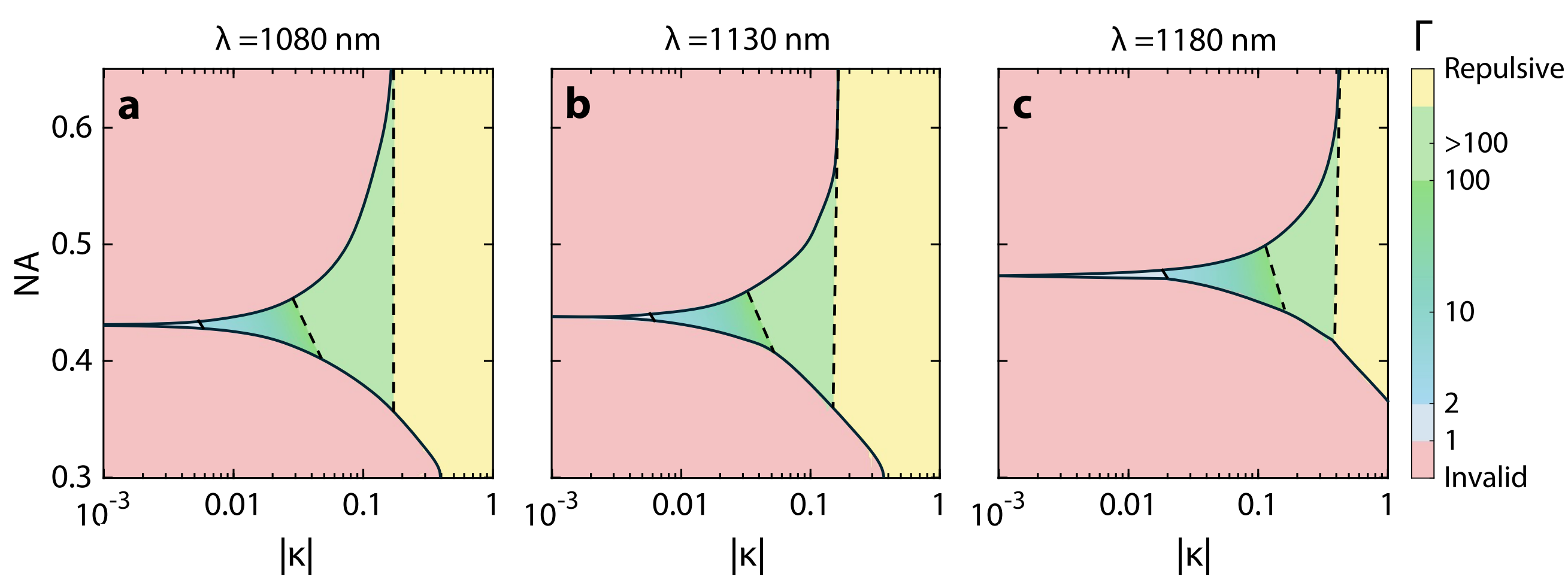

Figure S4: Trap selectivities, as depicted in Figure 5 of the main document, for a) 1080 nm, b) 1130 nm and c) 1180 nm. The illumination power here is set to 300 mW.

S5. Optical trap dynamics for higher power illuminations

The results shown in Figure 5 of the main document correspond to an illumination power of 100 mW, consistent with literature on enantioselective optical trapping. As noted there, the reddish region below the $|\kappa|$ minimum peak corresponds to situations where enantioselective optical trapping would be possible, but the trapping time would be insufficient (below 60 s). Such a limitation can be addressed with higher optical power, enlarging the achiral trapping potential and thus increasing the trapping time.

To assess the performance of our system under such conditions, we plot in Figure S4 a replica of Figure 5 with an illumination power of 300 mW. Since the mean escape time for the undesired enantiomer also grows, the whole profile of the selectivity shifts towards lower NA values. This could be beneficial since the normalized chiral difference $\Delta U_\kappa / U_0$ was larger at smaller NAs. However, operation at such powers should be considered with caution, since the strength of the fields might cause photothermal or photodisruptive damage to the samples.

S6. Heat generation in the nanodisks

Despite the low-loss nature of high refractive index dielectric materials such as silicon, the very high powers employed for optical trapping result in non-negligible temperature increments. To explore the thermal behavior of the system, stationary heat transfer simulations were carried out in COMSOL, using the absorbed electromagnetic power (resistive losses) in the silicon disks as the heat source. For silicon, a thermal conductivity of 148 W/(m·K) was considered, while 0.6 W/(m·K) and 1.4 W/(m·K) conductivities were considered for water and glass, respectively. An Interfacial Thermal Conductance (ITC) of 143 MW/(m^2·K) was considered between water and silicon [4].

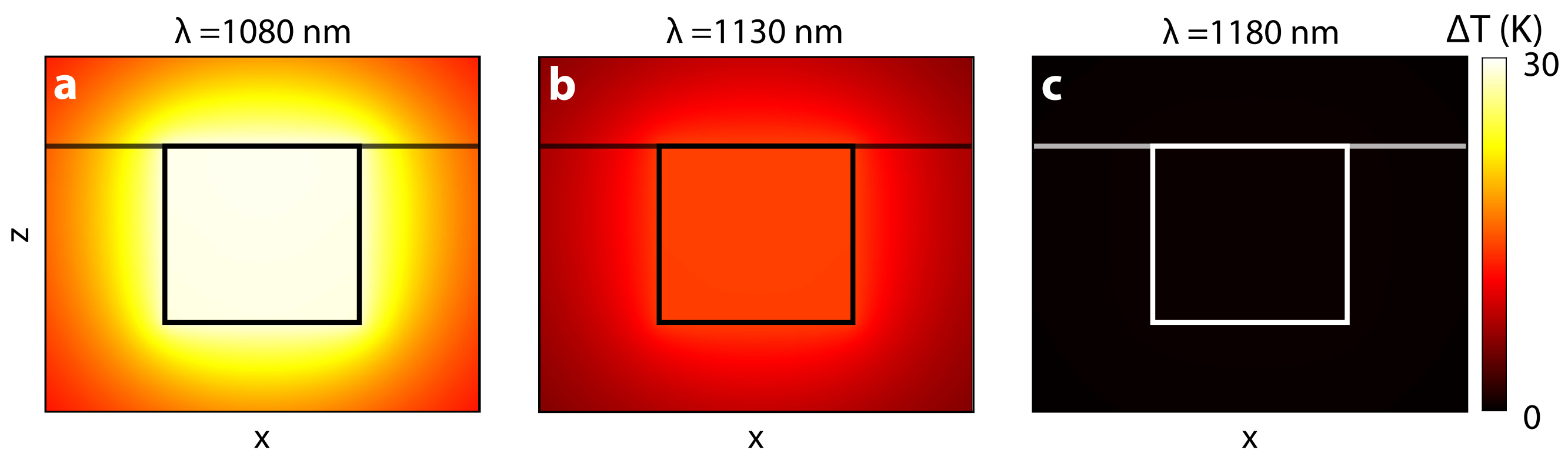

Figure S5: Temperature increment profiles for a) 1080 nm, b) 1130 nm and c) 1180 nm; at 100 mW illumination power and the maximum focusing strength considered ($NA = 0.64$).

Figure S5 shows the temperature profiles for the maximum focusing strength ($NA = 0.64$) at the cases examined in Figure 5 of the main document. In general, temperature increments are mild despite the relatively high power and tight focusing, as silicon losses are low in this spectral range. The temperature increase at the resonant wavelength (1130 nm) reaches around 15 K, while using lower wavelengths (1080 nm) can double the heating. Conversely, off-resonant wavelengths (1180 nm) result in negligible heating. It should be noted that temperature increments of ~ 20 K might result in permanent damage to some compounds (for example, denaturalization in proteins [5]), and thus, usage of larger disks, resonant at longer wavelengths where silicon losses are negligible, would be advisable. Another alternative would be to use dielectric materials with lower losses, such as GaP [6].

S7. Details on scattered-field simulations and comparison to full-field simulations

As stated in the methods section, numerical electromagnetic calculations in this work were performed in a scattered-field formulation in COMSOL (RF Module). To accurately solve this problem in the presence of a substrate, we introduce the background fields from equations S1 and S2 in a piecewise manner, following the constant refractive indices of glass ($n_G = 1.5$) and water ($n_W = 1.33$). To account for reflections and tranmissions, the piecewise fields were corrected by their corresponding Fresnel coefficients, shown in Figure S6. As the beam is emitted from the substrate side, the Fresnel coefficients consider glass as the first medium and water as the second.

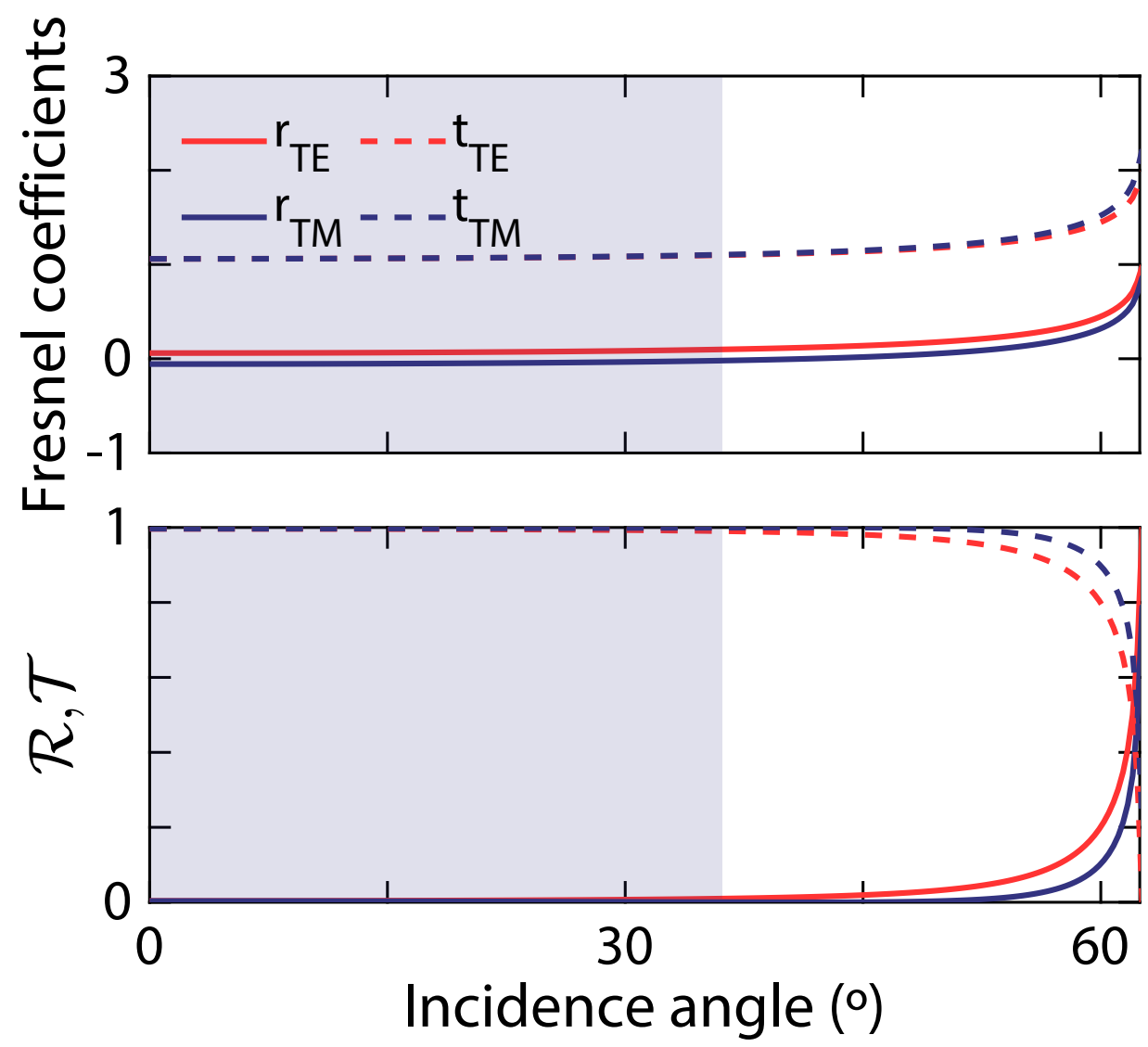

Figure S6: Fresnel coefficients (upper panel), as well as the reflection $\mathcal{R}$ and transmission $\mathcal{T}$ factors (lower panel) for both TE and TM polarizations. The shadowed area corresponds to the paraxial regime of the considered beams. For visualization clarity, only angles up to the critical angle $\theta_c = n_W/n_G \approx 62^{\text{o}}$ are displayed.

As shown in this image, the coefficients assume relatively constant values across the paraxial regime ($r_{TE} \approx 0.06$, $r_{TM} \approx -0.06$ and $t \approx 1.06$ for both polarizations). Thus, the corrections applied to the beams corresponded to these constant values for all simulations. It should be noted that the APB and RPB configurations correspond to TE and TM modes, and thus the TE coefficients were assigned to the azimuthal component of the field and the TM coefficients to the radial and longitudinal components.

To check the validity of our approach, we provide a comparison with full-field simulations in Figure S7. These full-field simulations were performed with Lumerical FDTD, employing its "Import source", where a custom field is injected at a single plane. We check two different situations: one with only the glass-water interface (Figure S7a-b) and another with both the interface and the $h = 240$ nm disk examined in Figures 1-2 of the main text (Figure S7c-d).

As shown in Figure S7, the agreement between the two methods in both cases is excellent, with the almost imperceptible differences being caused by meshing differences and/or different considerations of the silicon permittivity (in the case of simulations with the disk). It should be mentioned that a major factor in the accuracy of this approach resides in the tiny refractive index contrast between water and glass, which is responsible for the constant behaviour of the low-angle Fresnel coefficients. This can be observed from the transmission and reflection factors, where reflection is energetically negligible ($\mathcal{R}_{TE,TM} < 0.005$). Due to this, the shape of the field around the silicon disk (Figure S7)c-d) does not deviate heavily from the simulations in homogeneous media shown in Figure 1e and 1g in the main text.

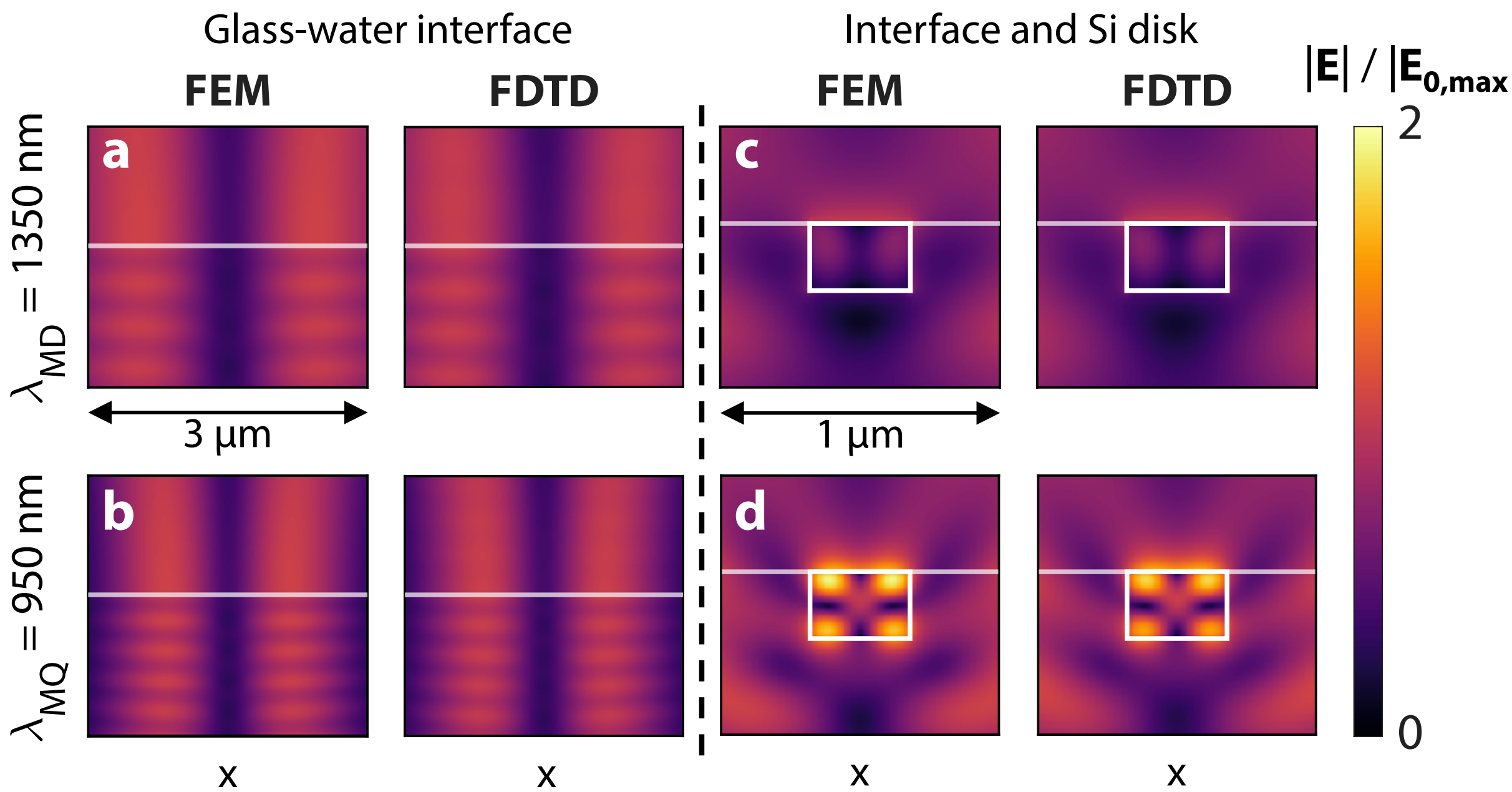

Figure S7: Transversal profiles (XZ planes) of the electric field magnitude obtained by our scattered-field approach in FEM simulations (COMSOL) and a full-field approach in FDTD (Lumerical). a) Simulations considering only the glass-water interface at the magnetic dipole (a) and magnetic quadrupole (b) resonance wavelengths in Figure 1. c) Simulations considering the glass-water interface plus the $h = 240$ nm silicon nanodisk at the magnetic dipole (c) and magnetic quadrupole (d) resonance wavelengths in Figure 1. Panels a and b display larger regions (3×3 μm images) to show the general profile of the beam, while panels c and d zoom in on the disk (1×1 μm images) for a better comparison of near-field features.

References

[1] A. Herrero-Parareda, F. Capolino, *Advanced Photonics Research* **2024**, *5* 2300265.

[2] A. B. Evlyukhin, T. Fischer, C. Reinhardt, B. N. Chichkov, *Physical Review B* **2016**, *94* 205434.

[3] P. H. Jones, O. M. Maragò, G. Volpe, *Optical Tweezers: Principles and Applications*, Cambridge University Press, 1st edition, **2015**.

[4] W. Gonçalves, M. Isaiev, D. Lacroix, S. Gomès, K. Termentzidis, *Surfaces and Interfaces* **2022**, *33* 102188.

[5] Y. Matsuura, M. Takehira, Y. Joti, K. Ogasahara, T. Tanaka, N. Ono, N. Kunishima, K. Yutani, *Scientific Reports* **2015**, *5* 1.

[6] D. E. Aspnes, A. A. Studna, *Physical Review B* **1983**, *27* 985.
